# Co-fermentation of yeast CECA and PL09 modulates antioxidant activity, volatile and metabolic profiles of highland barley bran

**DOI:** 10.1016/j.fochx.2026.104736

**Published:** 2026-10-31

**Authors:** Haotian Du, Yingrui Cao, Silin Li, Jingjing Feng, Jing Hao, Xiuzhu Yu, Shuangkui Du

**Affiliations:** a College of Food Science and Engineering, Northwest A&F University, 22 Xinong Road, Yangling, Shaanxi 712100, PR China; b Shaanxi Union Research Center of University and Enterprise for Grain Processing Technologies, Yangling, Shaanxi 712100, PR China; c Qinghai Xindingxiang Grain and Oil Co., Ltd., PR China

**Keywords:** Highland barley bran, Co-fermentation of yeast, Antioxidant activity, Volatile profiles, Non-volatile profiles

## Abstract

Highland barley bran (HBB) is a nutrient-rich by-product whose food application is limited by poor flavor and palatability. This study compared glucose-supplemented HBB fermented for 24 h with CECA, PL09, or their 1:1 mixed culture using HS-SPME-GC–MS and untargeted metabolomics. Among the fermented treatments, CP24 showed the highest total phenolic and flavonoid colorimetric indices, while its in vitro DPPH and ABTS radical-scavenging activities reached 30.37% and 54.98%, respectively. CP24 also exhibited a distinct VOC profile, with isoamyl acetate and ethyl hexanoate reaching eOAVs of 30.96 and 38.60, respectively, although their sensory contributions require validation. Metabolomics identified 42 and 227 differential metabolites relative to C24 and P24, and correlation analysis revealed statistical associations between selected metabolites and VOCs. These findings characterize the treatment-dependent chemical and metabolic differences in fermented HBB and support its further evaluation in cereal foods.

## 1 Introduction

Highland barley is an important cereal crop of the Qinghai–Tibet Plateau and is valued for its diverse bioactive constituents (Chen, Zhu, et al., 2023; Du et al., 2022; Obadi et al., 2021). Its processing generates substantial amounts of highland barley bran (HBB), which is rich in dietary fiber, arabinoxylans, phenolic acids, and other valuable components but remains underutilized (Wang et al., 2024; Zhang, Li, et al., 2024). The coarse texture and undesirable flavor of HBB, together with the limited bioaccessibility of compounds bound within the cell-wall matrix, restrict its wider application in food products (Fall et al., 2025; Mu et al., 2024; Qin et al., 2025; Xi et al., 2023). Recent studies have shown that fungal fermentation can alter the nutritional quality, physicochemical properties, antioxidant activity, and volatile composition of HBB, while optimized solid-state fermentation can promote polyphenol release and enhance antioxidant activity (Zhang, Liu, et al., 2024; Zhang, Li, et al., 2024). These studies have established the general feasibility of using microbial fermentation to modify HBB, although their emphasis has mainly been placed on overall quality changes or the optimization of fermentation conditions for specific physicochemical and antioxidant indices. Less attention has been given to how different yeast inoculation modes influence both the volatile composition and non-volatile metabolome of HBB under directly comparable conditions, or to the statistical associations between these two groups of metabolites. Clarifying these differences could provide a more specific basis for selecting yeast fermentation strategies for the value-added utilization of HBB.

Fermentation, as a traditional and efficient food processing method, can alter the abundance and distribution of substrate components through microbial metabolism (Ashaolu et al., 2025; Bai et al., 2024; Wang et al., 2026). Previous studies have demonstrated that fermentation can modulate carbohydrate and amino acid metabolism in cereals and their by-products, facilitate the release of bound bioactive compounds, and influence the formation and accumulation of volatile organic compounds (VOCs) (Niu et al., 2025; Saha et al., 2024). In cereal bran, these changes are often accompanied by reductions in undesirable green and rancid notes (Chen et al., 2025). The specific effects of fermentation are closely related to the metabolic characteristics of the microorganisms involved. Filamentous fungi generally secrete a range of extracellular enzymes that facilitate the breakdown of complex bran matrices. Lactic acid bacteria can modify substrate composition through acidification, phenolic biotransformation, and the production of organic acids and other metabolites. Yeasts, in turn, contribute to the formation of alcohols, esters, aldehydes, and other volatile compounds through carbohydrate, amino acid, and lipid metabolism (Ashaolu et al., 2025; Freire et al., 2015; Sun et al., 2025; Wei et al., 2024). In addition to their role in aroma formation, yeasts may facilitate the release and transformation of bound bioactive compounds and reshape the composition of low-molecular-weight metabolites (Qin et al., 2025; Tiwari et al., 2025). Given these differences in microbial metabolism, mixed-culture fermentation has increasingly been explored for cereal-bran modification. For example, yeast–lactic acid bacteria co-culture fermentation has been shown to alter the aroma profile, phenolic composition, and antioxidant activity of wheat bran (Tang et al., 2024). These findings confirm the application of mixed-culture fermentation to cereal bran while also indicating that its metabolic outcomes vary with the microbial combination, substrate characteristics, and culture conditions. For HBB, integrating volatile and non-volatile metabolite analyses may reveal metabolic differences among fermentation modes, although their sensory and nutritional implications require further validation.

Strain selection is important because strain-specific metabolic characteristics may lead to differences in fermentation performance and metabolite composition (Ashaolu et al., 2025). *Saccharomyces cerevisiae* is widely used in cereal- and plant-based fermentations because of its robust fermentative capacity and broad substrate adaptability (Parapouli et al., 2020). CECA is an indigenous *S. cerevisiae* strain with favorable fermentation kinetics and aroma-forming potential, particularly in relation to floral and fruity aroma characteristics (Zhang et al., 2023). PL09 belongs to the non-*Saccharomyces* yeast *Torulaspora delbrueckii* and is characterized by its ability to produce esters and glycerol while maintaining relatively low levels of volatile acidity and hydrogen sulfide (Liu et al., 2022). Its metabolic characteristics differ from those of *S. cerevisiae*, providing a rationale for comparing the two yeasts in monoculture and mixed-culture fermentations. However, the effects of CECA and PL09, used individually or as a mixed inoculum, on the volatile and non-volatile metabolic profiles of HBB have not been systematically compared under identical substrate and culture conditions. In particular, the relationships between non-volatile metabolites and changes in VOCs across these fermentation modes remain unclear.

Accordingly, individual fermentation of CECA and PL09 and co-fermentation of them for HBB were compared in this study to to improve HBB for its potential development as a nutrient-enhancing powdered ingredient for liangpi, dough-based products, and other cereal foods. The effects of different fermentation modes on volatile flavor characteristics and small-molecule metabolic profiles have been investigated by headspace solid phase microextraction gas chromatography–mass spectrometry (HS-SPME-GC–MS), and non-targeted metabolomics. Partial least squares discriminant analysis (PLS-DA) was used to reveal the key differential metabolites (DMs) and their pathways in the co-fermentation of HBB by different yeast. Pearson correlation coefficient was performed to reveal the correlation of key volatile organic compounds (VOCs) and DMs. These results would provide theory evidence for the co-fermentation of yeast PL09 and CECA regulating the flavor characteristics and small-molecule metabolic profiles of HBB.

## 2 Materials and methods

### 2.1 Materials

Highland barley (*Hordeum vulgare* L. var. nudum) were obtained by Qinghai Xindixiang Grain and Oil Co., Ltd. (Qinghai, China). The crude bran fraction was obtained from highland barley using an LSM20 experimental mill, and then dried at 40 °C for 24 h, ground, and passed through a 60-mesh sieve to obtain the HBB sample. The yeast strains of PL09 and CECA were purchased from Shanghai Dingtang International Trade Co., Ltd. (Shanghai, China). PL09 (commercial name: Viniflora® Prelude) was a commercial active dry preparation of the non-*Saccharomyces* yeast *Torulaspora delbrueckii* manufactured by Chr. Hansen A/S (Denmark). According to the manufacturer's technical specifications, PL09 has a relatively slow fermentation rate, an optimal fermentation temperature of 20–25 °C, an ethanol tolerance of approximately 9–10% (*v*/v), glycerol production of approximately 5–8 g/L, and low production of volatile acidity and hydrogen sulfide. Its taxonomic identity and technological characteristics were documented according to the manufacturer's technical information. PL09 is the commercial designation of the strain, and no public culture-collection accession number was provided by the manufacturer. CECA (commercial name: CN-Style CECA) was a commercial active dry preparation of *Saccharomyces cerevisiae* manufactured by Angel Yeast Co., Ltd. (China). According to the manufacturer's technical information, CECA was selected from the Ningxia wine-producing region by a research team led by Professor Yanlin Liu at the College of Enology, Northwest A&F University. It has a moderate fermentation rate, a suitable fermentation temperature range of 15–35 °C, and an ethanol tolerance of up to 17% (*v*/v). CECA is also characterized by low foam formation, high flocculation, low volatile acidity, and very low hydrogen sulfide production. The taxonomic identities, origins, and technological characteristics of both yeasts were documented according to the manufacturers' technical information. PL09 and CECA are commercial designations, and no publicly accessible culture-collection accession numbers were provided by the manufacturers. Potato Dextrose Agar (PDA) was purchased from Qingdao Hope Bio-Technology Co., Ltd. (Qingdao, China).

### 2.2 The preparation of fermented HBB

Fermented HBB was prepared according to a previously reported method with slight modifications (Bertsch et al., 2020). In brief, 40 g of HBB was placed in a 250-mL Erlenmeyer flask and mixed with 100 mL of deionized water at a solid-to-liquid ratio of 1:2.5 and ultrasonicated for 20 min, followed by autoclaving at 121 °C for 20 min. After cooling, 8 mL of sterile 25% (*w*/*v*) glucose solution was aseptically added to each HBB substrate, including the uninoculated control. This solution contained 2 g of glucose, equivalent to 5% (*w*/w) of the dry HBB mass. Glucose was added as a standardized and readily fermentable carbon source to support yeast growth and the initiation of fermentation in the bran substrate. Thereafter, the sterilized HBB substrate was then inoculated with 12 mL of CECA suspension, 12 mL of PL09 suspension, or 12 mL of a 1:1 mixture of the CECA and PL09 suspensions (6 mL of each suspension) to achieve an initial inoculation concentration of approximately 10^6^ CFU/mL. For the uninoculated control, 12 mL of sterile diluent was added instead of the yeast suspension, ensuring that the initial amounts of added glucose and liquid were identical among all treatments. The uninoculated HBB was named as CK, while HBB fermented with CECA, PL09, and their mixed culture were named as C, P, and CP, respectively. All treatments were incubated statically at 25 °C for 24 h without agitation or forced aeration. Oxygen availability was therefore determined mainly by the flask headspace and the passive gas exchange permitted by the flask closure. Relative humidity was not independently controlled because fermentation was conducted in flasks containing the hydrated HBB substrate rather than in an open incubation system. After incubation, the corresponding samples were designated as CK, C24, P24, and CP24, respectively. The samples were pre-frozen at −80 °C for 24 h and then freeze-dried. The freeze-dried samples were stored at 4 °C for further analysis.

### 2.3 Determination of bioactive components and antioxidant capacity of fermented HBB

Firstly, The extract was carried out according to previous reported method with some modifications (Li, Luo, et al., 2024). Briefly, 0.1 g of HBB was extracted with 2.5 mL of 60% ethanol under ultrasonic treatment at 300 W, with 5 s working intervals and 8 s pauses, at 60 °C for 30 min. The extract was then centrifuged at 12,000 rpm and 25 °C for 10 min, and the supernatant was collected and adjusted to 2.5 mL with 60% ethanol for further analysis.

#### 2.3.1 Determination of total phenolic content

The total phenolic content of fermented HBB were measured based on the method described by Zugazua-Ganado et al. (2024) with some modifications. Firstly, volume 100 μL of the above extract were fully mixed with 3 mL of distilled water and 200 μL of Folin–Ciocalteu reagent, and then stand for 5 min. Thereafter, 800 μL of 10% (*W*/*V*) Na_2_CO_3_ solution were added and reacted for 1 h. Finally, above reaction were measured at 765 nm with a UV spectrophotometer. The total polyphenol content was expressed as gallic acid equivalent (mg/g).

#### 2.3.2 Determination of total flavonoid content

The *total flavonoid content* were measured according to the method described by Shraim et al. (2021) with slight modifications. Briefly, 100 μL of sample was mixed with 0.2 mL of 5% (*w*/*v*) NaNO₂ solution and vortexed. After 5 min, 0.2 mL of 10% (*w*/*v*) Al(NO₃)₃ solution was added and the mixture was allowed to react for 6 min. Then, 2.0 mL of 4% (*w*/*v*) NaOH solution was added and the reaction proceeded at room temperature for 15 min. The absorbance was measured at 510 nm using a UV–Vis spectrophotometer (1 cm path length). A reagent blank (without sample) was prepared in parallel. Results were expressed as mg rutin equivalents per g of sample (mg RE/g).

#### 2.3.3 Determination of antioxidant capacity

Prior to the measurement of antioxidant capacity, the sample extract was performed as follows. Briefly, 0.5 g of freeze-dried fermented HBB powder was mixed with 10 mL of 70% ethanol and extracted by ultrasonic treatment for 30 min at room temperature. The mixture was then centrifuged at 8000 *g* for 10 min, and the supernatant was collected as the sample extract. The antioxidant capacity of fermented HBB was evaluated using commercial ABTS and DPPH radical scavenging assay kits (Cat. No. UPLC-W-ABTS and UPLC-W-DPPH, respectively) according to the manufacturer's instructions. For the ABTS assay, the sample extract was mixed with the corresponding reagents and incubated at room temperature in the dark for 6 min, after which the absorbance was measured at 405 nm. For the DPPH assay, the sample extract was mixed with the DPPH working solution and incubated at room temperature in the dark for 30 min, and the absorbance was measured at 515 nm. The radical scavenging activities were calculated according to the kit instructions and expressed as percentages.

### 2.4 Determination of VOCs in fermented HBB by HS-SPME-GC–MS

VOCs from different fermented HBB were extracted using headspace solid-phase microextraction (HS-SPME). Briefly, frozen samples were taken from −80 °C storage and ground under liquid nitrogen. After vortex mixing, approximately 500 mg of each sample was weighed into a headspace vial. Subsequently, 2 mL of saturated NaCl solution and 20 μL of internal standard solution (10 μg/mL) were added. The samples were then subjected to automated HS-SPME extraction prior to GC–MS analysis. The extraction was performed at 60 °C with shaking for 5 min, followed by headspace extraction using a 120 μm DVB/CWR/PDMS SPME Arrow fiber for 15 min. The analytes were desorbed at 250 °C for 5 min. Before extraction, the fiber was conditioned at 250 °C for 5 min in a Fiber Conditioning Station, while a new fiber was conditioned for 2 h before use.

GC–MS analysis was performed using a DB-5MS capillary column (30 m × 0.25 mm × 0.25 μm; Agilent J&W Scientific, Folsom, CA, USA). High-purity helium (≥99.999%) was used as the carrier gas at a constant flow rate of 1.2 mL/min. The injector temperature was maintained at 250 °C, and the solvent delay time was 3.5 min. The oven temperature program was as follows: 40 °C held for 3.5 min, increased to 100 °C at 10 °C/min, then increased to 180 °C at 7 °C/min, and finally increased to 280 °C at 25 °C/min and held for 5 min. Mass spectrometry was conducted using an electron impact ionization source. Full-scan data were first acquired for chromatographic peak detection and tentative compound annotation, whereas selected ion monitoring (SIM) mode was subsequently used for selective detection and semiquantitative analysis of the predefined qualitative and quantitative ions. The ion source, quadrupole, and interface temperatures were set at 230 °C, 150 °C, and 280 °C, respectively. The electron energy was 70 eV, and qualitative and quantitative ions were monitored according to the precise scanning method described in GB 23200.8-2016.

Following peak detection and extraction from the full-scan data, the resulting chromatographic features were annotated by comparing their mass-spectral fingerprints, including the qualitative and quantitative ions, with reference spectra in the NIST 17 library and by additionally comparing their retention indices with reference RI values for non-polar chromatographic columns. Based on the full-scan data and the established volatile-compound database, the retention times and characteristic qualitative and quantitative ions of the candidate VOCs were determined and subsequently monitored in SIM mode. Thus, the reported VOCs were not discovered or identified solely from SIM data; full-scan data were used for tentative annotation, whereas SIM data were used for selective detection and semiquantification. The Match factor represents the combined score derived from mass-spectral fingerprint matching and RI comparison. The quantitative ions, qualitative ions, reference RIs, and Match factors of the individual compounds are provided in Table S1. Because authentic standards were not analyzed under the same analytical conditions, the compounds were regarded as tentatively identified VOCs (Level 2 putatively annotated compounds) rather than unequivocally confirmed compounds. Accordingly, none of the reported VOCs were assigned to Level 1 identification.

The semiquantitative concentration of each VOC was estimated from its peak area relative to that of the internal standard. Because compound-specific calibration curves and response factors were not established, the reported concentrations should be interpreted as semiquantitative estimates rather than absolute concentrations. The water-based odor thresholds were obtained from van Gemert (2011), as specified for the individual compounds in Table 1. The estimated odor activity value (eOAV) was obtained by dividing the concentration of each VOC by its reported odor threshold (OT) in water. Because the concentrations were semiquantitative and the odor thresholds were obtained in water rather than in the HBB matrix, the calculated eOAVs were used only as a preliminary screening indicator. Compounds with eOAVs equal to or greater than 1 were regarded as candidate aroma-active VOCs that might contribute to the aroma characteristics of the samples, rather than as compounds with confirmed sensory importance (Xiong et al., 2023).

**Table 1:** eOAVs (*n* = 3) of aroma-active compounds with eOAV ≥1 identified aroma components in fermented HPP.

Compounds	Class	aOT (μg/L)	beOAV				Odor Quality	Possible contribution to CP flavor
			CK	C24	P24	CP24		
5-Ethyl-3-hydroxy-4-methyl-2(5H)-furanone	Ketone	40	17.40 ± 0.08b	19.30 ± 0.40ab	20.85 ± 1.49a	21.25 ± 0.93a	sweet, fruity, caramel, maple, nutty, butterscotch	Sweet, fruity and caramel-like notes; may enhance aroma richness and pleasant sweetness in CP.
1-(2-Aminophenyl)ethanone/ 2-aminoacetophenone	Ketone	2	4.33 ± 0.49b	7.22 ± 0.82a	3.58 ± 0.31c	7.56 ± 0.20a	grape, sweet	Higher in CP than other groups; may contribute grape-like and sweet aroma.
2-Nonenal	Aldehyde	0.25	5.74 ± 0.22b	6.95 ± 0.34b	6.28 ± 0.62b	8.08 ± 0.47a	green, waxy, cucumber, melon	May strengthen green, cucumber and melon-like freshness in CP.
2-Hexenal	Aldehyde	17	3.9 ± 0.13b	4.03 ± 0.07ab	4.13 ± 0.24ab	4.5 ± 0.05a	sweet, almond, fruity, green, apple	May contribute sweet, fruity, green and apple-like notes.
3-Methylbutyl acetate / isoamyl acetate	Ester	2	0.6 ± 0.02d	23.4 ± 1.37b	3.68 ± 0.59c	30.96 ± 0.43a	sweet, fruity, banana, green, ripe	Markedly higher in CP; likely supports banana-like, ripe fruit and sweet aroma.
Ethyl decanoate	Ester	5	0.39 ± 0.30b	30.75 ± 3.67a	21.32 ± 7.12a	30.98 ± 9.76a	sweet, fruity, apple, grape, brandy	May enhance sweet, fruity, apple/grape and brandy-like aroma.
Pentanal	Aldehyde	12	0.47 ± 0.47c	28.21 ± 4.61a	9.92 ± 0.99b	29.92 ± 4.5a	fermented, bread, fruity, nutty, berry	Higher in CP; may contribute fermented, fruity, nutty and berry-like notes.
Ethyl 3-methylbutanoate / ethyl isovalerate	Ester	0.1	2.83 ± 0.10a	3.01 ± 0.13a	3.12 ± 0.26a	3.43 ± 0.08a	strawberry, candy, fruity	May contribute strawberry, candy-like and fruity aroma.
4-Heptanone	Ketone	8.2	3.66 ± 0.10a	3.75 ± 0.08a	3.76 ± 0.20a	3.87 ± 0.03a	fruity, cheese, sweet, cognac, pineapple	May add fruity, sweet, cognac and pineapple-like notes.
Ethyl hexanoate	Ester	5	0.28 ± 0.02d	33.5 ± 1.77b	4.17 ± 0.46c	38.6 ± 0.65a	apple, pear, fruity	Markedly higher in CP; likely contributes apple, pear and fruity aroma.

a OT: odor threshold (μg/L).

b eOAV: odor activity value. eOAVs were recalculated after semi-quantification with the internal standard: concentration = peak area of compound / peak area of internal standard × 400 μg/kg, where 400 μg/kg was derived from 10 μg/mL internal standard × 20 μL / 0.5 g sample.

The compounds listed here are those with eOAV ≥1 and their odor qualities are mainly sweet, fruity, green, floral, caramel-like or fresh notes.

### 2.5 Analysis of non-volatiles of fermented HBB

Fermented HBB samples were freeze-dried in a vacuum freeze dryer (Scientz-100F) for 63 h and then ground into powder using a grinder (MM 400, Retsch) at 30 Hz for 1.5 min. Approximately 30 mg of sample powder was accurately weighed and extracted with 1500 μL of 70% methanol aqueous internal standard extraction solution pre-cooled at −20 °C. For samples weighing less than 30 mg, the extraction solvent was added at a ratio of 1500 μL per 30 mg of sample. The internal standard extraction solution was prepared by dissolving 1 mg of standard compound in 1 mL of 70% methanol aqueous solution to obtain a 1000 μg/mL stock solution, which was further diluted with 70% methanol to prepare a 250 μg/mL internal standard solution. During extraction, the samples were vortexed once every 30 min for 30 s each time, for a total of six times. After centrifugation at 12,000 rpm for 3 min, the supernatant was collected, filtered through a 0.22 μm microporous membrane, and transferred into injection vials for subsequent UPLC-MS/MS analysis.

Untargeted metabolomic analysis was performed using a UPLC-MS/MS system. Chromatographic separation was carried out on a Waters ACQUITY UPLC HSS T3 column (1.8 μm, 2.1 mm × 100 mm). Mobile phase A consisted of ultrapure water containing 0.1% formic acid, and mobile phase B consisted of acetonitrile containing 0.1% formic acid. The column temperature was maintained at 40 °C, the flow rate was set at 0.40 mL/min, and the injection volume was 4 μL. Mass spectrometric data were acquired using a Q Exactive HF-X mass spectrometer.

The raw MS data were first converted into mzML format using ProteoWizard, and peak extraction, alignment, and retention time correction were then performed using the XCMS program. In the present study, a metabolic feature was defined as an LC-MS signal characterized by a specific retention time, precursor-ion mass-to-charge ratio (*m*/*z*), and ion intensity. Such features were distinguished from putatively annotated metabolites because the assignment of a candidate compound name does not constitute definitive identification. Peaks with a missing rate greater than 50% among samples in each group were filtered out, and missing values were imputed. Features with blank values greater than 50% were filled with one-fifth of the minimum value, whereas features with blank values less than 50% were imputed using the K-nearest neighbor method. Peak areas were subsequently corrected using the support vector regression method.

The corrected metabolic features were identified by searching an in-house database, integrated public databases, predicted databases, and the metDNA approach. Candidate annotations were assigned by jointly considering the measured precursor-ion *m*/*z*, theoretical accurate mass, mass error, retention time, adduct type, molecular formula, database-matching score, and available database records. Nevertheless, database-assisted annotation does not provide unequivocal compound identification in the absence of confirmation with authentic standards. Finally, metabolic features with an integrated annotation score greater than 0.5 and a coefficient of variation below 0.5 in the QC samples were retained. Data from positive and negative ion modes were then merged, and for features assigned repeatedly to the same candidate metabolite, the annotation with the highest confidence level and integrated annotation score was retained for subsequent statistical analysis.

The retained annotations were classified according to the confidence-level system of the analytical platform, with Level 2 representing relatively higher-confidence putative compound annotations and Level 3 representing lower-confidence candidate compound or compound-class annotations. Because no authentic standards were analyzed under the same analytical conditions, none were considered Level 1 identifications. Accordingly, the 1764 retained features, comprising 359 Level 2 and 1405 Level 3 annotations, are described as putatively annotated metabolic features rather than definitively identified metabolites. Their precursor-ion *m*/*z*, molecular formula, retention time, adduct type, mass error, annotation level and score, chromatographic mode, and available database identifiers are provided in Table S2. Peak areas were used only to compare relative changes in the same feature across samples or treatments and not for absolute quantification or direct comparisons among different metabolites.

For each pairwise comparison, *P* values were adjusted across all metabolic features subjected to statistical testing using the Benjamini–Hochberg procedure to control the false discovery rate (FDR). Differential metabolites were screened using VIP ≥ 1.0, |log₂(fold change)| ≥ 1, and FDR-adjusted *P* < 0.05.

### 2.6 Statistical analysis

Each treatment (CK, C24, P24, and CP24) comprised three independently prepared biological replicates (*n* = 3), with each biological replicate obtained from a separate fermentation flask. All experimental measurements and instrumental analyses were performed using these three biological replicates. The results were expressed as the mean ± standard deviation (mean ± SD). Significant differences among different treatment groups were analyzed by one-way analysis of variance, followed by Duncan's multiple range test. Differences were considered statistically significant at *p* < 0.05. Peak extraction, peak filtering, retention time correction, and peak alignment were then performed using the XCMS package in R to generate a metabolite quantitative matrix. Subsequent data analysis and figure generation were conducted using the online cloud platform of Suzhou Panomics Biotechnology Co., Ltd., MetaboAnalyst 4.0, and Origin 2021. Heatmaps were generated using TBtools-II software (v2.052).

## 3 Results and discussion

### 3.1 The total phenolic and total flavonoid content of fermented HBB

HBB is rich in phenolic and flavonoid compounds, many of which occur in bound forms within the cell-wall matrix, limiting their extractability and bioaccessibility (He et al., 2023). As shown in Fig. 1a-b, yeast fermentation increased the total phenolic content (TPC) and total flavonoid content (TFC) of HBB. Specifically, the TPC increased from 2.55 mg/g DW in CK to 2.85 mg/g DW in C24 and 2.76 mg/g DW in P24, respectively, and reached the highest level of 2.92 mg/g DW in CP24. Similarly, the TFC increased from 2900 μg/g DW in CK to 3114 and 3029 μg/g DW in C24 and P24, respectively, and further increased to 3147 μg/g DW in CP24. Thus, all three fermentation treatments increased the TPC and TFC of HBB to varying degrees, with CP24 showing the highest levels at 24 h. Previous studies have suggested that microbial fermentation may improve the extractability of phenolic and flavonoid compounds by modifying the bran cell-wall structure and disrupting their linkages with macromolecular components (Adebo & Gabriela Medina-Meza, 2020; Gao et al., 2025). These changes might explain the higher contents of TPC and TFC, although cell-wall degradation, enzyme activity, and bound-compound release were not directly measured. Prabhu et al. (2016) also reported that *Saccharomyces cerevisiae* fermentation enhanced the total phenolic and flavonoid contents of wheat bran increased by 34% and 20%, respectively. The relatively high TPC and TFC of CP24 might be associated with differences in substrate utilization and metabolic activity under mixed-culture conditions.

**Fig. 1:**
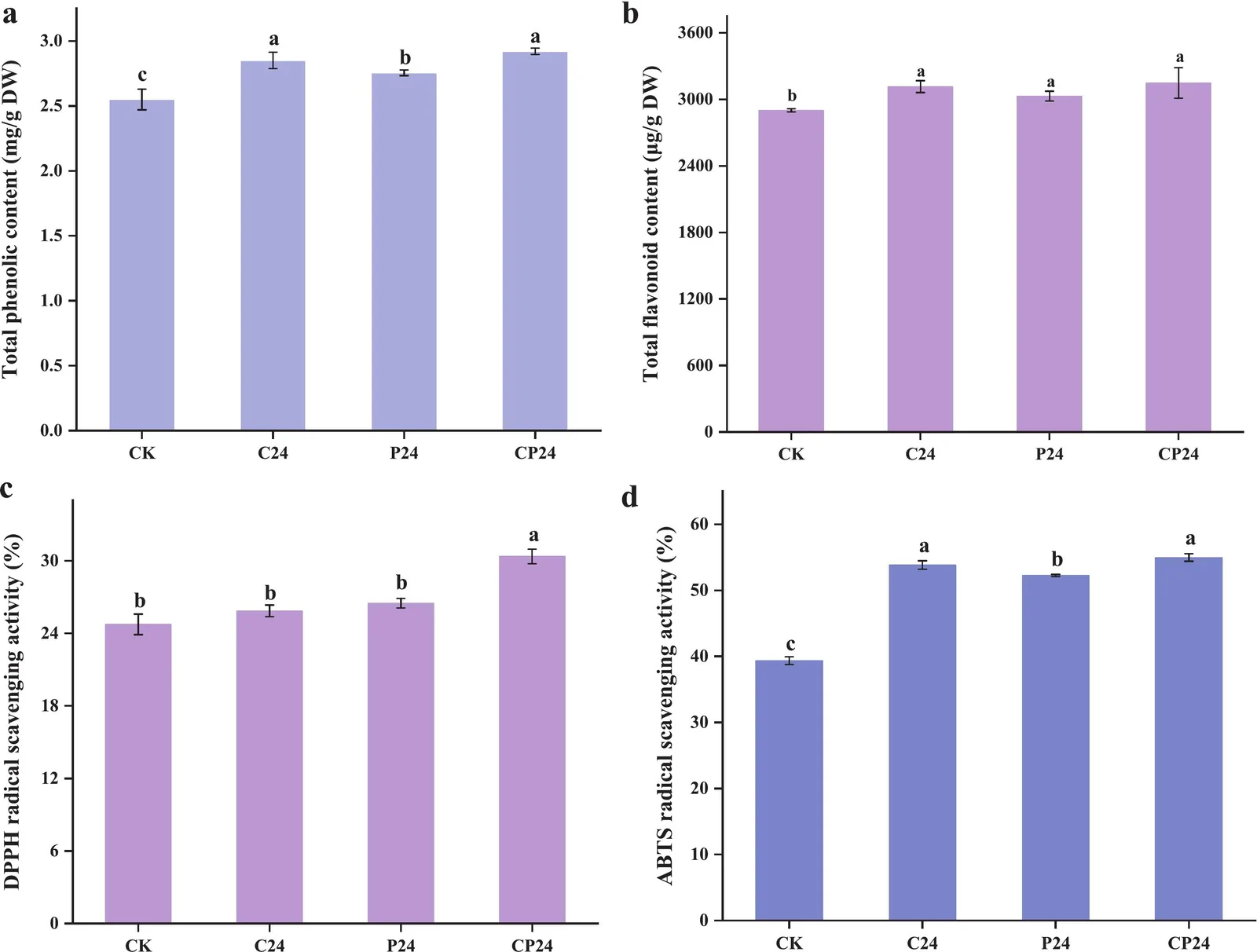
Total phenolic content (a), total flavonoid content (b), DPPH and ABTS radical scavenging activities of HBB during PL09, CECA, co-fermentation of them (c-d).

Although the Folin–Ciocalteu and total flavonoid colorimetric assays are widely used for comparative analysis of fermented cereal samples, their analytical limitations should be considered (Chen et al., 2025). The Folin–Ciocalteu reagent can react with some non-phenolic reducing substances, while the total flavonoid assay may be affected by flavonoid structure and matrix components (Shraim et al., 2021). Because all samples were analyzed under identical conditions, the results remain useful for relative comparisons among groups but should be interpreted as colorimetric estimates expressed as equivalents of the corresponding standards. Future studies will use targeted HPLC or LC–MS analysis with authentic standards to further validate changes in individual phenolic and flavonoid compounds.

### 3.2 The antioxidant capacity of fermented HBB

DPPH and ABTS radical scavenging capacities are important indicators for evaluating the antioxidant properties of fermented products. As shown in the Fig. 1c-d, the DPPH radical scavenging activity increased from 24.74% in CK to 25.87% and 26.50% in C24 and P24, respectively, and reached the highest level of 30.37% in CP24 (Fig. 1c). CP24 exhibited the highest DPPH radical-scavenging activity, representing an increase of 5.63% over CK. This result suggested that mixed-culture fermentation enhanced the DPPH radical-scavenging capacity of HBB to some extent. The ABTS radical scavenging activity increased from 39.36% in CK to 53.85% and 52.24% in C24 and P24, respectively, and further increased to 54.98% in CP24 (Fig. 1d). Most of the increase in ABTS radical-scavenging activity occurred after monoculture fermentation, with CP24 exceeding C24 and P24 by only 1.13% and 2.74%, respectively, indicating a limited additional increase from mixed-culture fermentation. The changes in radical-scavenging activity after fermentation may be related to alterations in multiple extractable components of the samples (Adebo & Gabriela Medina-Meza, 2020). Phenolic and flavonoid compounds are rich in active groups such as hydroxyl groups, which can exert antioxidant effects through hydrogen atom donation, electron transfer, or direct scavenging of free radicals (Chiorcea-Paquim & Oliveira-Brett, 2023; Leopoldini et al., 2011). Accordingly, the changes in TPC and TFC shown in Fig. 1a–b may be associated, at least in part, with the observed variations in radical-scavenging activity. Organic acids, amino acid derivatives, and other metabolites whose abundances differed among treatments may also contribute to the observed radical-scavenging activities (Yu et al., 2026). Overall, CP24 showed the highest in vitro DPPH and ABTS radical-scavenging activities, although its additional increase in ABTS activity over the monoculture treatments was small. These chemical assay results warrant further investigation of the biological antioxidant effects of fermented HBB.

### 3.3 The flavor characteristics of fermented HBB

#### 3.3.1 Composition analysis of VOCs of fermented HBB

The composition and abundance of VOCs provide an important chemical basis for understanding aroma-related changes in fermented HBB, although their actual sensory contributions also depend on factors such as odor thresholds and interactions within the food matrix. A total of 970 VOCs were detected and annotated across the four groups, including 183 esters, 150 terpenoids, 122 ketones, 104 alcohols, 61 aldehydes, 54 acids, and other compounds (Fig. 2a and Table 1). Overall, the VOC profiles of the three inoculated groups differed from that of CK, and the extent of these differences varied with the inoculated strain or strain combination. Tang et al. (2024) showed that co-fermentation of *S. fibuligera* with several LAB strains altered the volatile profile of wheat bran, particularly its ethyl esters, and further evaluated the sensory effects in bran-containing Chinese steamed bread. In contrast, the present study was limited to the chemical characterization of fermented HBB without sensory or product-level validation. In the present HBB fermentation system, CP24 had a higher total VOC content than C24 and P24 after 24 h of fermentation (Table S1). Compared with C24 and P24, CP24 showed a distinct VOC distribution (Fig. 2b-c), which showed higher enrichment in esters, ketones, and aldehydes (Fig. 2d). Based on odor characteristics previously reported for the detected compounds, CP24 contained relatively high abundances of VOCs associated with sweet, fruity, and green notes (Fig. 2e-h). Strain-dependent metabolism might contribute to these differences in VOC profiles (Dzialo et al., 2017). Overall, CP24 exhibited a distinct volatile composition, particularly in the ester, ketone, and aldehyde fractions.

**Fig. 2:**
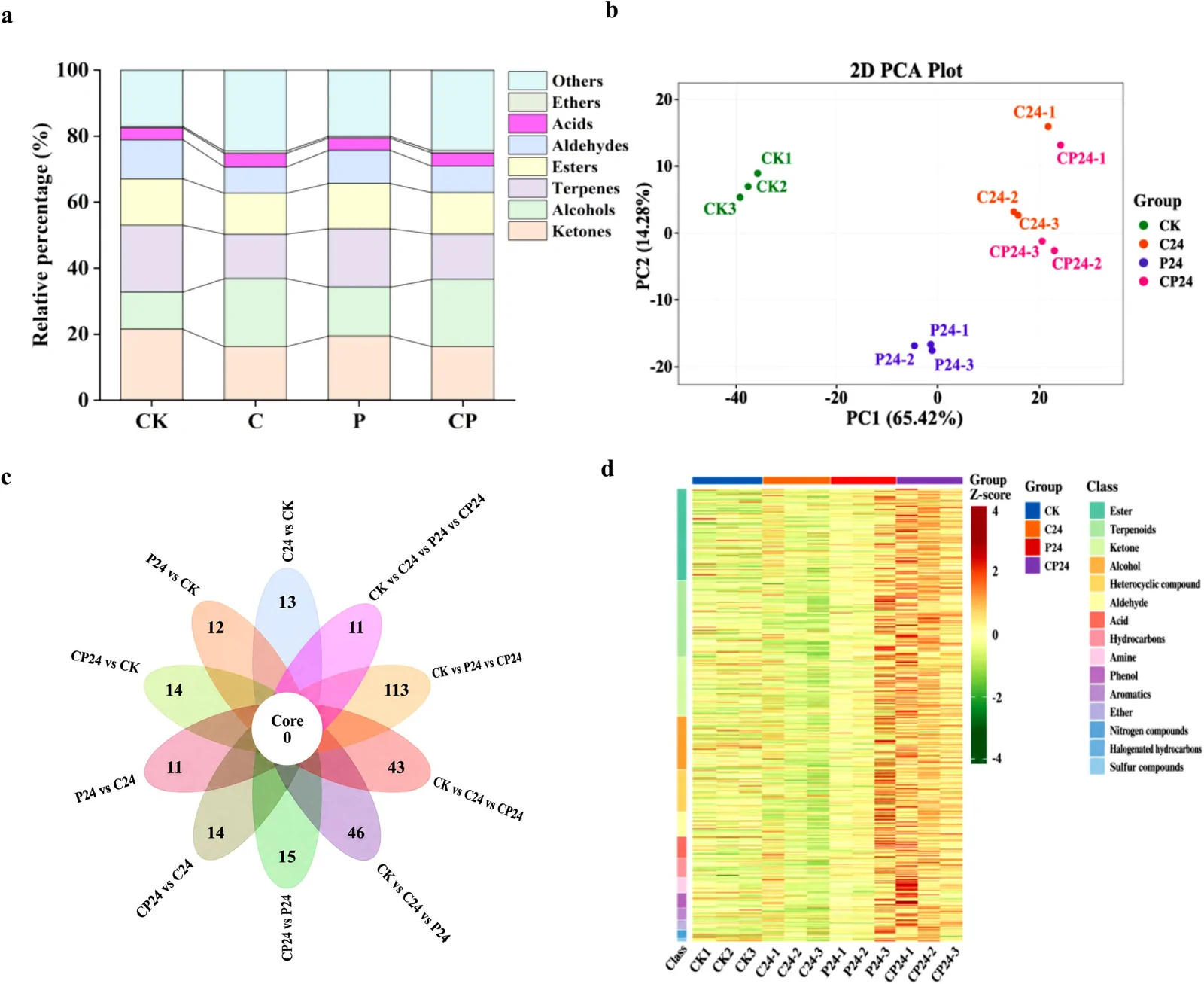
The volatile compounds of HBB after yeast fermentation of 24 h. Sacked distribution chart of volatile compounds in different samples (a), PCA plots of CK, C24, P24, and CP24 samples (b), Venn diagram of common and unique metabolites between different comparison groups (c), Heatmap clustering of volatile compounds base on *p*-values (d).

Alcohols were among the relatively abundant VOC classes in fermented HBB. Depending on the compound, they can be associated with alcoholic, floral, grassy, fatty, or mildly sweet notes (Chen, Li, et al., 2023). Alcohols can also serve as substrates for ester formation during fermentation. In general, The total alcohol content increased from 10,508.24 μg/kg in CK to 30,814.99 μg/kg in C24 and 18,218.26 μg/kg in P24, reaching the highest level of 32,882.64 μg/kg in CP24 (Table S1). The alcohol compounds detected in all samples mainly include 1-butanol, 2-pentanol, 1-butanol, 2-octanol, 3-octanol, 4-heptanol, and 2,6-dimethyl (Table S1). Among them, 2-pentanol increased sharply from 397.20 μg/kg in CK to 5799.36 μg/kg in C24, 2252.59 μg/kg in P24, and 6191.61 μg/kg in CP24. Similarly, (*S*)-2-methyl-1-butanol increased from 324.47 μg/kg in CK to 4930.19 μg/kg in C24, 1838.35 μg/kg in P24, and 5148.99 μg/kg in CP24. The greater accumulation of these alcohols in C24 and CP24 than in P24 was associated with the presence of CECA under the current culture conditions. Yeast sugar and amino acid metabolism, together with the use of alcohols as precursors for ester biosynthesis, provides a possible context for these compositional differences (Liu et al., 2023). Overall, C24 and CP24 showed higher total alcohol contents and higher levels of these two alcohols than P24.

Ester compounds can contribute fruity, sweet, and ripe-fruit notes, depending on their concentrations and odor thresholds (Gao et al., 2025). During yeast fermentation of HBB, several esters, including acetate esters and ethyl esters, showed higher measured levels than in CK (Table S1). The total ester content increased from 13,060.55 μg/kg in CK to 18,481.11 μg/kg in C24 and 16,836.17 μg/kg in P24, and reached the highest value of 20,181.74 μg/kg in CP24 (Table S1). Yeast ester biosynthesis involves alcohol and acyl-CoA precursors associated with amino acid and fatty acid metabolism (Dzialo et al., 2017), but the pathways underlying the observed differences were not directly examined here. In particular, ethyl 3-octenoate increased from 2908.69 μg/kg in CK to 3108.52 μg/kg in C24, 3519.61 μg/kg in P24, and 3473.00 μg/kg in CP24. Ethyl octanoate was almost absent in CK, with a content of only 2.97 μg/kg, but increased markedly to 1194.10 μg/kg in C24 and 1231.63 μg/kg in CP24. Similarly, ethyl decanoate increased from 1.94 μg/kg in CK to 452.16 μg/kg in C24 and 453.93 μg/kg in CP24. Although CP24 had the highest total ester content, individual esters showed different responses. Ethyl 3-octenoate was slightly higher in P24, whereas ethyl octanoate and ethyl decanoate accumulated mainly in CECA-containing treatments. Thus, CP24 exhibited a distinct overall ester profile without consistently exceeding the monocultures in individual ester levels. These differences may have implications for fruity and sweet aroma characteristics, although sensory verification is needed.

Aldehyde compounds can be associated with green, grassy, woody, cucumber-like, and fruity odor notes, depending on the compound (Saha et al., 2024). Several aldehydes showed increased levels after HBB fermentation, such as pentanal, 2-hexenal, and 2-nonenal (Table S1). The total aldehyde content increased from 11,096.20 μg/kg in CK to 11,978.93 μg/kg in C24, 12426.78 μg/kg in P24, and 13,003.09 μg/kg in CP24. Among them, (E)-2-hexenal increased from 2649.23 μg/kg in CK to 2746.42 μg/kg in C24, 2799.67 μg/kg in P24, and 3059.73 μg/kg in CP24. A similar trend was observed for 2-hexenal, which increased from 2647.34 μg/kg in CK to 3057.42 μg/kg in CP24. Because many aldehydes have relatively low odor thresholds, even moderate changes in their concentrations may influence the overall balance of aroma-related attributes (Xiong et al., 2023). Although aldehydes can undergo conversion to alcohols or acids during fermentation (Sun et al., 2025), such conversions were not established in the present study. Overall, CP24 showed the highest total aldehyde content, contributing to its distinct chemical VOC profile. The odor descriptions reported for these compounds suggest potential green and fruity notes, which require sensory validation.

Ketones and terpenoids also contributed to the differences in volatile composition among the fermented HBB samples. Some compounds within these classes are commonly associated with sweet, fruity, creamy, caramel-like, floral, and fresh odor characteristics. The total ketone content increased from 19,631.29 μg/kg in CK to 23,843.62 μg/kg in C24 and 23,553.38 μg/kg in P24, and reached the highest value of 25,847.97 μg/kg in CP24 (Table S1). Carbohydrate, amino acid, and fatty acid metabolism might provide a biochemical context for these changes (Xiong et al., 2023). The detected terpenoid compounds included geraniol, citronellol, linalool, and α-curcumene (Table S1). Previous studies have suggested that yeast-associated enzymatic activity may release or convert terpenoid aroma precursors (Chen, Li, et al., 2023). Overall, the higher total ketone content in CP24 and the occurrence of terpenoid-associated VOCs further characterized the volatile composition of fermented HBB.

Overall, CECA, PL09, and their mixed culture produced distinct VOC profiles in HBB at 24 h. CP24 showed relatively high total levels of several VOC classes, whereas individual compounds responded differently to the three fermentation treatments, suggesting that each inoculation mode shaped the volatile composition of HBB in a distinct manner. All comparisons were conducted under the same glucose-supplemented conditions, allowing the differences among treatments to reflect the distinct metabolic outcomes of CECA, PL09, and their mixed culture within a standardized fermentation system. Nevertheless, glucose availability may influence yeast growth and the formation of alcohols, esters, organic acids, and other metabolites (Visinoni et al., 2022). Therefore, the added glucose may have contributed to the accumulation of some VOCs, making it difficult to distinguish its contribution from that of endogenous HBB components. Moreover, VOC formation and depletion are dynamic processes, and some compounds may follow non-linear patterns during fermentation. Because only the 24 h endpoint was examined, the present results represent a snapshot of the metabolic state at that time and cannot resolve the temporal relationships among substrate utilization, changes in non-volatile metabolites, and the accumulation of alcohols, organic acids, esters, and other VOCs. Thus, the proposed biochemical explanations remain hypotheses based on endpoint measurements and previous studies. Future time-course studies tracking key substrates and metabolites, together with quantitative determination of key odorants, odor activity value analysis, and sensory evaluation, are needed to clarify changes in VOC composition and their actual aroma contributions during fermentation.

#### 3.3.2 Identification of discriminant and potentially aroma-active VOCs using PLS-DA and eOAV analysis

The VOC profiles varied among the HBB fermentation treatments (Fig. 2 and Fig. 3a-c). To identify the VOCs primarily responsible for distinguishing these treatments, PLS-DA models were constructed using the VOC abundance data, and variable importance in projection (VIP) scores were used to evaluate the contribution of individual VOCs to group separation. Model validation was performed using 200 permutation tests for the CP24 vs C24 and CP24 vs P24 comparisons (Fig. S1). The original models yielded R^2^Y and Q^2^ values of 1.000 and 0.966 for CP24 vs C24, and 0.996 and 0.961 for CP24 vs P24, respectively. For both comparisons, the empirical permutation *p*-values for R^2^Y and Q^2^ were < 0.005, indicating that the original models outperformed the permuted models in terms of fit and predictive performance. A VIP score > 1 indicates that a variable contributes substantially to discrimination among groups, but does not necessarily indicate that it contributes strongly to perceived aroma (Shao et al., 2024). A total of 17 VOCs with VIP scores >1 were identified across the comparisons, including 5-ethyl-3-hydroxy-4-methyl-2(5H)-furanone, 2-aminoacetophenone, (E)-2-nonenal, 2-nonenal, (E)-2-hexenal, 2-hexenal, isoamyl acetate, ethyl decanoate, pentanal, ethyl 3-methylbutanoate, 4-heptanone, ethyl hexanoate, 2-methylbutyl acetate, pentanoic acid, 2,6-dimethyl-4-heptanol, 2-hexanethiol, and 2-pentenoic acid (Fig. 3d-f). These compounds collectively characterized the differences in VOC composition among the fermentation treatments, although their contributions to perceived aroma cannot be established from VIP scores alone.

**Fig. 3:**
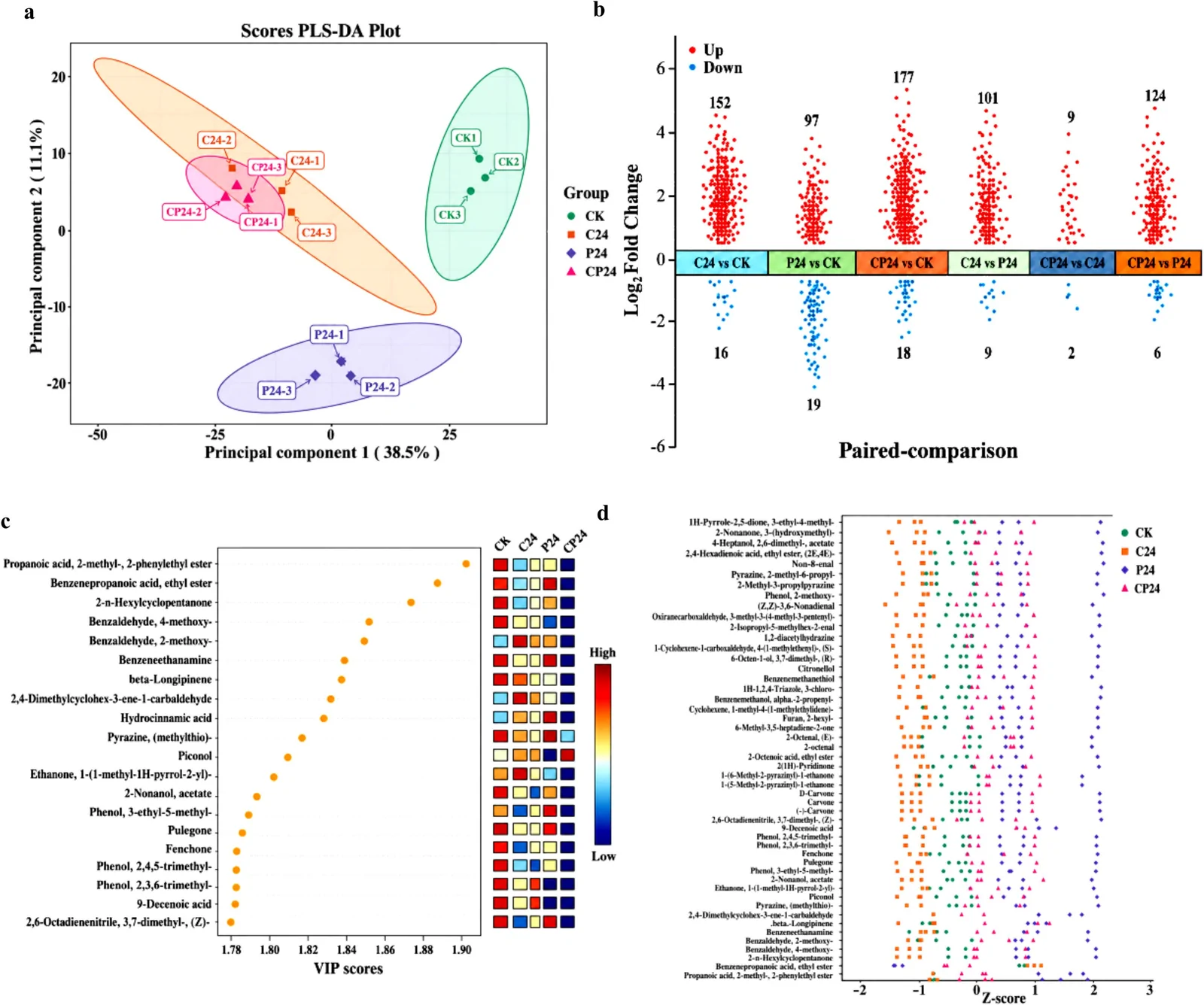
Characteristic volatile compounds of different fermented HBB. The scatter plot of Partial least squares discrimination analysis (PLS-DA) (a), The numbers labeled on each comparison represented the amounts of up- or down-regulated volatile compounds (b), *Z*-score plot of differential volatile compounds (c-d).

To further evaluate their potential aroma relevance, eOAVs were calculated from the measured concentrations and the corresponding odor thresholds reported previously, Compounds with eOAVs ≥1 are generally considered potentially aroma-active because their concentrations exceed their reported odor thresholds. However, because odor thresholds may vary among food matrices, the calculated eOAVs were used here as screening indicators rather than direct measures of sensory contribution. A total of 10 compounds with eOAVs ≥1 were screened as potentially aroma-active, including5-ethyl-3-hydroxy-4-methyl-2(5H)-furanone, 2-aminoacetophenone, 2-nonenal, 2-hexenal, isoamyl acetate, ethyl decanoate, pentanal, ethyl 3-methylbutanoate, 4-heptanone, and ethyl hexanoate, etc. Among these compounds, the aldehydes 2-hexenal, 2-nonenal, and pentanal are commonly associated with fresh, grassy, woody, and fruity odor notes (Table 1). The esters isoamyl acetate, ethyl hexanoate, ethyl decanoate, and ethyl 3-methylbutanoate are generally associated with fruity, sweet, and ripe-fruit notes. The furanone 5-ethyl-3-hydroxy-4-methyl-2(5H)-furanone and the ketones 2-aminoacetophenone and 4-heptanone have been associated with sweet, caramel-like, fruity, and grape-like odor characteristics. These compounds therefore represent candidate odorants for further sensory investigation, rather than confirmed contributors to the aroma of fermented HBB.

Compared with CK, C24 and P24, CP24 showed higher concentrations and eOAVs for several of these potentially aroma-active compounds (Table 1). In particular, isoamyl acetate and ethyl hexanoate exhibited the highest eOAVs in CP24, reaching 30.96 and 38.60, respectively, which were significantly higher than those in C24 and P24 (*P* < 0.05). Their relatively high eOAVs, together with previously reported fruity odor descriptions, identified them as candidate odorants for sensory evaluation of CP24. Ethyl decanoate also showed a high eOAV in CP24 (30.98), comparable to C24 (30.75), indicating a similar estimated aroma relevance in both CECA-containing treatments. In addition, pentanal and 2-nonenal showed higher eOAVs in CP24, reaching 29.92 and 8.08, respectively, which were significantly higher than those in C24 and P24 (*P* < 0.05). Together, these results characterized the distinct eOAV profile of CP24 at the 24 h endpoint and highlighted compounds for further sensory investigation. Nevertheless, their higher concentrations and eOAVs do not by themselves demonstrate synergistic interactions between CECA and PL09 or an improvement in overall sensory quality.

Some discriminant VOCs had eOAVs <1 and were therefore unlikely to contribute strongly as individual compounds at the measured concentrations. Such compounds may still influence the overall aroma through additive, suppressive, or other interactions, but these effects cannot be established from eOAVs alone. Moreover, because only the 24 h fermentation endpoint was examined, the PLS-DA and eOAV results reflect differences in VOC profiles among treatments at that specific time and cannot reveal their dynamic formation patterns or temporal relationships with non-volatile metabolites. Further time-course analyses, combined with GC–O and sensory evaluation, are needed to verify the changes in these candidate VOCs and their actual contributions to aroma.

### 3.4 Non-volatile compounds of fermented HBB

#### 3.4.1 The impact of yeast fermentation on the metabolites of HBB

Untargeted metabolomics was further used to characterize differences in non-volatile metabolite profiles among the HBB fermentation treatments. As shown in Fig. 4a, 1764 untargeted metabolites were detected in all samples, which could be classified into 20 categories. Among them, amino acids and their derivatives (17.53%), benzene and substituted derivatives (14.09%), alcohol and amines (8.22%), alkaloids (7.38%), phenolic acids (4.19%), and lipids (3.86%) were the main categories. These percentages describe the distribution of annotated metabolites among chemical classes rather than their relative concentrations or nutritional contributions. Several of these classes include compounds potentially relevant to antioxidant activity and flavor formation. For example, amino acids can serve as precursors for certain VOCs through the Ehrlich pathway and other metabolic routes (Wang et al., 2026). However, differences in amino acid abundance alone do not establish increased precursor conversion, improved nutritional value, or a causal relationship with the TPC and TFC. Alcohols and amines likewise encompass compounds that may participate in microbial metabolism and flavor-related reactions (Chen, Li, et al., 2023; Wang et al., 2026). Their abundance patterns here describe compositional differences without directly demonstrating their formation, consumption, or effects on yeast growth. Phenolic acids and lipids may also be relevant to antioxidant activity and volatile composition, although their individual contributions were not determined in this study. The metabolic profiles of samples from different groups showed significant differences (Fig. 4b-c). Compared with CK, C24, P24, and CP24 exhibited different metabolite abundance profiles under the glucose-supplemented fermentation conditions. CP24 also showed a distinct profile relative to C24 and P24 (Fig. 4c), highlighting differences between mixed-culture and monoculture fermentation. Overall, the treatments yielded distinct non-volatile metabolite profiles, with CP24 differing from both monoculture treatments at the 24 h endpoint.

**Fig. 4:**
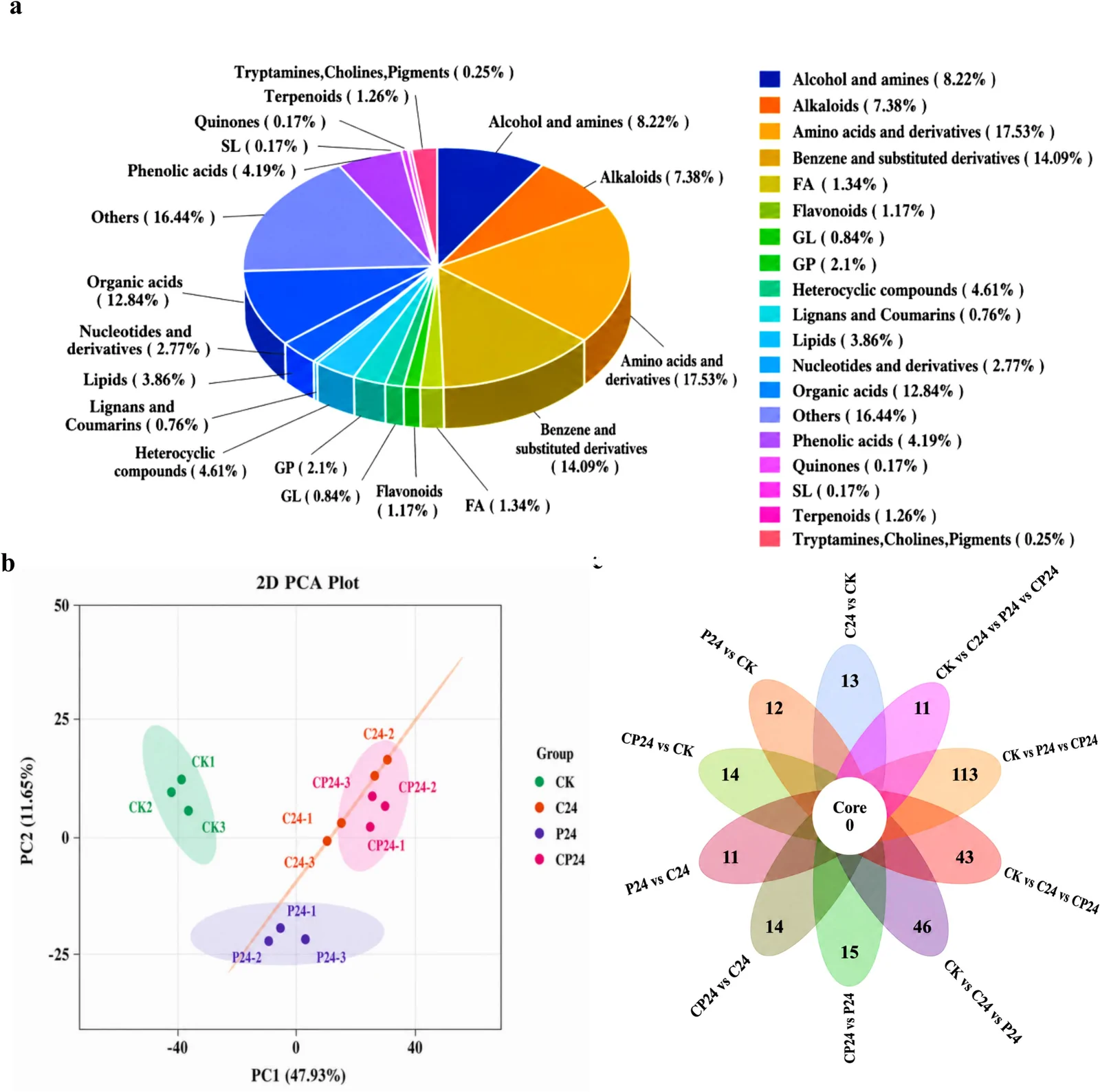
Characteristics of non-volatile metabolites of different fermented HBB. Classification of all detected metabolites of fermented HBB (a), Partial least squares discrimination analysis (PLS-DA) plot (b), Venn diagram of common and unique metabolites between different comparison groups (c). The numbers labeled on each comparison represented the amounts of up- or down-regulated metabolites.

#### 3.4.2 Differential metabolites analysis between fermented HBB

The metabolic differences between the monoculture and mixed-culture treatments were examined using pairwise PLS-DA models. As shown in Fig. 5a and 6a, CP24 was separated from C24 and P24 in the corresponding PLS-DA score plots, indicating treatment-dependent differences in their non-volatile metabolic profiles. VIP scores were obtained from the respective PLS-DA models and combined with fold-change analysis and FDR-adjusted *P* values to identify differential metabolites. Metabolites meeting all three criteria of VIP ≥ 1.0, |log₂(fold change)| ≥ 1, and FDR-adjusted *P* < 0.05 were classified as differential metabolites (DMs). Based on these criteria, 42 DMs were identified between CP24 and C24, while 227 DMs were identified between CP24 and P24 (Fig. 5b–c and 6b–c). Compared with C24, CP24 showed higher relative abundances of several representative DMs, including 2,4,8-trimethylsphingoic acid isobutyrate, 4-methyloctanoic acid, Glc4Me(a1–4)Glc(a)-O-Me, and isopentanol (Fig. 5d–e). According to their chemical classifications and database annotations, these metabolites represented sugar derivatives, fatty acids, alcohols, and other chemical categories (Niu et al., 2025). These differences further characterized the distinct metabolic profile of CP24 at the 24 h endpoint but did not directly demonstrate enhanced pathway activity, precursor release, or nutritional improvement. Compared with P24, the levels of 4-methyloctanoic acid, isocitric acid, *N*-acetyl-*D*-phenylalanine, and 9-(2-deoxypentofuranosyl)-2- [(2-methylacryloyl)amino]-9H-purine-6-alcohol in CP24 were obviously increased (Fig. 6d-e). These annotations encompassed fatty acids, organic acids, amino acid derivatives, and nucleotide-related compounds (Sun et al., 2025). Their abundance differences indicate that mixed-culture fermentation produced a chemical profile distinct from PL09 monoculture, without establishing increased substrate conversion or utilization. In particular, the higher relative abundance of *N*-acetyl-*D*-phenylalanine highlighted a difference in an aromatic amino acid derivative. Although amino acid metabolism can contribute to yeast aroma formation (Dzialo et al., 2017), this observation alone does not demonstrate enhanced pathway activity or greater availability of aroma precursors. Overall, CP24 differed from both monoculture treatments across several metabolite classes. These findings characterize the mixed-culture treatment at 24 h, while the interactions between CECA and PL09 underlying these differences remain to be determined.

**Fig. 5:**
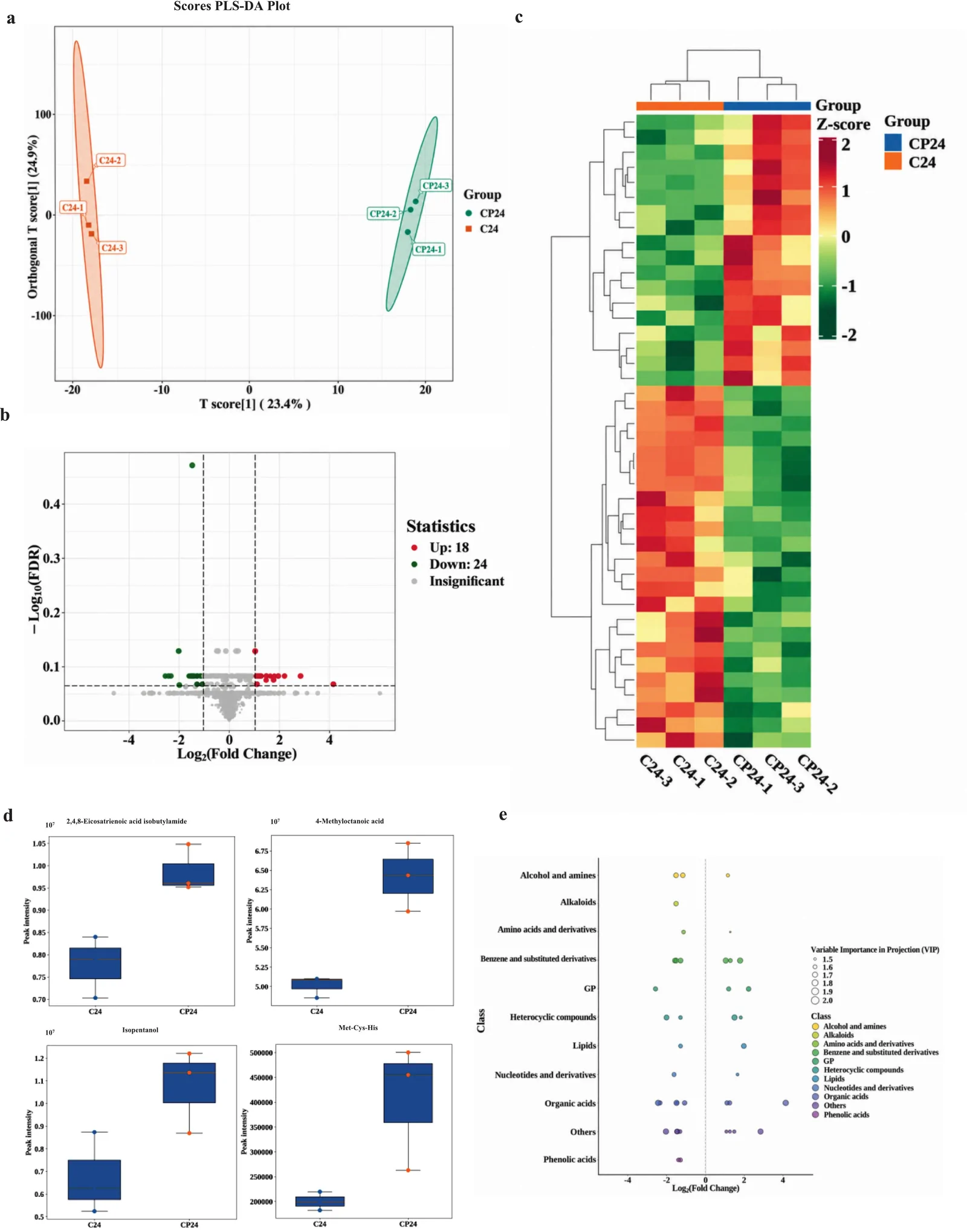
Metabolic profile difference in the HBB fermented 24 h with individual yeast strain CECA (C24) and both of yeast strain CECA and yeast strain PL09 (CP24). The scatter plot of Partial least squares discrimination analysis of C24 and CP24 (a), Volcanic plot of DMs distribution between C24 and CP24 (b), Heat map of DMs between C24 and CP24 (c), The columns represent samples and the rows represent metabolites and the red and green colors indicate relatively high and low expression, respectively, Box plots of the top 4 identified DMs base on *p*-values (d), Bubble plot of differential metabolite classes (e). (For interpretation of the references to colour in this figure legend, the reader is referred to the web version of this article.)

**Fig. 6:**
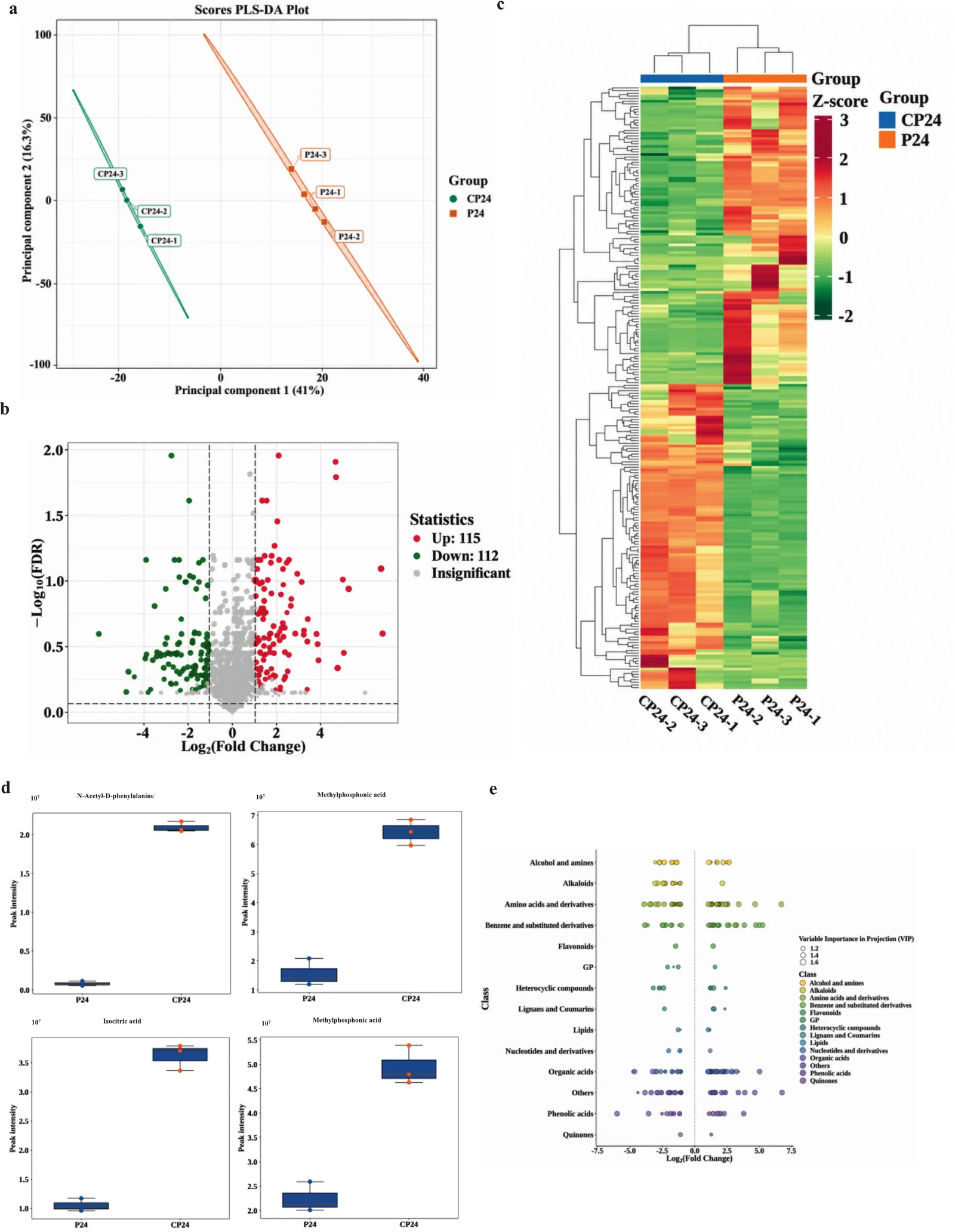
Metabolic profile difference in the HBB fermented 24 h with individual yeast strain PL09 (P24) and both of yeast strain CECA and yeast strain PL09 (CP24). The scatter plot of Partial least squares discrimination analysis of P24 and CP24 (a), Volcanic plot of DMs distribution between P24 and CP24 (b), Heat map of DMs between P24 and CP24 (c), The columns represent samples and the rows represent metabolites and the red and green colors indicate relatively high and low expression, respectively, Box plots of the top 4 identified DMs base on *p*-values (d), Bubble plot of differential metabolite classes (e). (For interpretation of the references to colour in this figure legend, the reader is referred to the web version of this article.)

### 3.5 The correlation analysis of key VOCs and DMs

Pearson correlation analysis was performed to explore statistical associations between key VOCs and DMs in fermented HBB (Fig. 7a). The key VOCs, including isoamyl acetate, ethyl decanoate, and 2,6-dimethyl-4-heptanol, were positively correlated with several key DMs, including 2,4,8-trimethylsphingoic acid isobutyrate, 4-methyloctanoic acid, and *N*-acetyl-*D*-phenylalanine. These correlations indicate that the selected VOCs and DMs showed similar patterns of variation among treatments. In addition, several key DMs including acetophenone, D-ribulose, sucrose, and 3-hydroxytetradecanoic acid were negatively correlated with selected VOCs. These negative correlations indicate opposing patterns of variation among the fermentation treatments, but they do do not establish that these metabolites were consumed as substrates or converted into aroma-active compounds. Based on their annotations, D-ribulose and sucrose are sugars, 3-hydroxytetradecanoic acid is a hydroxy fatty acid, and acetophenone is an aromatic ketone. These associations therefore link differences in several non-volatile metabolite classes with variation in VOC profiles, without identifying specific metabolic pathways or reaction directions. Previous studies have also reported links between sugar, fatty acid, amino acid, and organic acid metabolism and VOC formation in other fermented products (Li, Xu, et al., 2024; Niu et al., 2025; Sun et al., 2025).

**Fig. 7:**
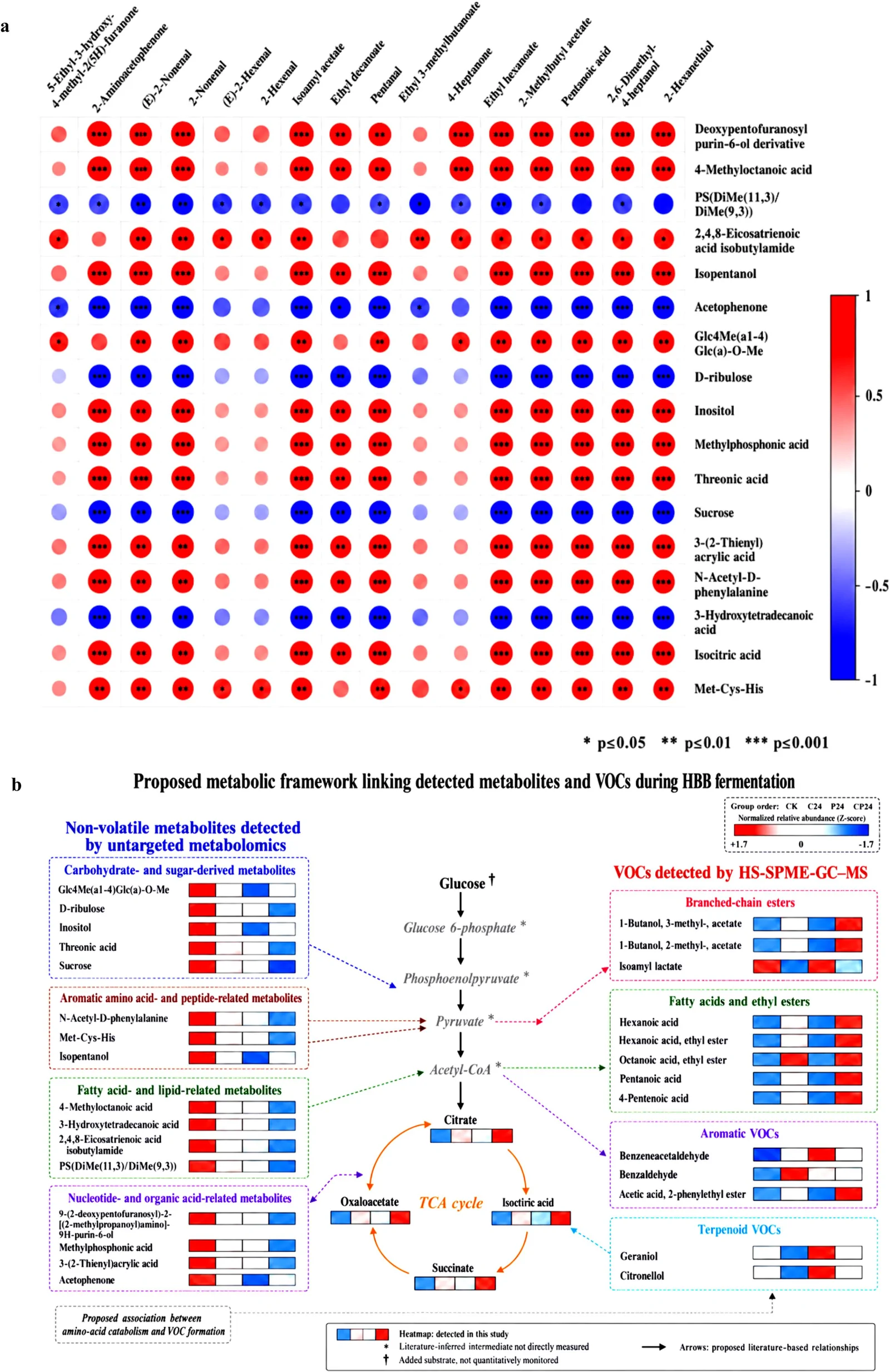
The correlation analysis of key volatile compounds and non-volatile compounds based on Pearson coefficient (a) and the important metabolic pathways of HBB during yeast fermentation (b).

Based on these results, a hypothetical metabolic model was developed to illustrate the potential links between metabolic changes and VOC formation in CP24 by integrating the observed changes in differential metabolite abundance, correlation analysis, and current knowledge of yeast metabolism (Fig. 7b). Changes in carbohydrate-related metabolites, including sucrose, D-ribulose, and inositol, might be associated with central carbon metabolism and linked to amino acid/peptide, fatty acid/lipid, and organic acid metabolism through key intermediates such as phosphoenolpyruvate, pyruvate, and acetyl-CoA. These interconnected pathways might provide a metabolic basis for the formation of alcohols, aldehydes, ketones, acids, and esters. In particular, changes in amino acid-, fatty acid-, and organic acid-related metabolites might be associated with differences in fruity ester VOCs, including ethyl hexanoate, ethyl decanoate, and isoamyl acetate. This model was constructed primarily from the metabolic changes and statistical associations observed in this study, together with previously reported biochemical knowledge. Therefore, it is intended to provide a plausible framework for interpreting differences in VOC composition among the fermentation treatments rather than to establish specific precursor–product relationships or metabolic fluxes.

## 4 Conclusion

This study systematically compared the effects of CECA, PL09, and their 1:1 mixed culture on the chemical and metabolic characteristics of glucose-supplemented HBB at the 24 h endpoint. Compared with the single-strain treatments, CP24 showed higher total phenolic and flavonoid colorimetric indices and greater activity in the in vitro antioxidant assays. CP24 also exhibited a distinct VOC profile, with higher concentrations and eOAVs of several potentially aroma-active compounds, particularly isoamyl acetate and ethyl hexanoate. These compositional differences provide a chemical basis for potential fruity and sweet aroma-related characteristics but do not demonstrate improved sensory quality or consumer acceptance. Untargeted metabolomics further revealed treatment-dependent differences in metabolic profiles, while correlation analysis identified statistical associations between selected VOCs and metabolites related to carbohydrate, amino acid/peptide, fatty acid/lipid, and organic acid metabolism. These associations offer hypotheses for interpreting the observed VOC variations but do not establish specific precursor–product relationships or metabolic pathways. Overall, the findings provided insight into the chemical and metabolic differences between single-strain and mixed-culture fermentation of HBB. This study offer a basis for exploring fermented HBB as a cereal-food ingredient, with future trials in products such as liangpi, dough, and bread needed to evaluate its processing performance, sensory acceptability, and nutritional bioavailability.

## CRediT authorship contribution statement

**Haotian Du:** Writing – review & editing, Writing – original draft, Visualization, Methodology, Investigation, Formal analysis. **Yingrui Cao:** Writing – review & editing, Writing – original draft, Methodology, Formal analysis. **Silin Li:** Writing – original draft. **Jingjing Feng:** Writing – original draft. **Jing Hao:** Writing – original draft. **Xiuzhu Yu:** Supervision, Data curation, Conceptualization. **Shuangkui Du:** Supervision, Project administration, Investigation, Funding acquisition, Formal analysis, Data curation, Conceptualization.

## Declaration of competing interest

The authors declare that they have no known competing financial interests or personal relationships that could have appeared to influence the work reported in this paper.

## Data Availability

Data will be made available on request.

## Appendix A Supplementary data

Supplementary material: Additional tables and figures related to the volatile and metabolic profiles of highland barley bran co-fermented by yeast CECA and PL09.
