# Supplementary material for: Co-fermentation of yeast CECA and PL09 modulates antioxidant activity, volatile and metabolic profiles of highland barley bran
Source: Food Chem X. 2026 Oct 31;40:104736. doi: 10.1016/j.fochx.2026.104736 (PMC13745735; doi:10.1016/j.fochx.2026.104736)
Supplement: Supplementary file 1 [file mmc1.docx]

**Co-fermentation of** **yeast CECA and PL09 improves the flavor and functional characteristics of highland barley bran**

Haotian Du ^a^, Yingrui Cao ^a^, Silin Li ^a^, Jingjing Feng ^a^, Jing Hao ^a, c^，Xiuzhu Yu ^a, b^, Shuangkui Du ^a, b, [1]*^

^a^ *College of Food Science and Engineering, Northwest A&F University, 22 Xinong Road, Yangling, Shaanxi 712100, PR China*

^b^ *Shaanxi Union Research Center of University and Enterprise for Grain Processing Technologies, Yangling, Shaanxi 712100, PR China*

^c^ *Qinghai Xindingxiang Grain and Oil Co., Ltd.*

**A**

**B**

**Figure S1. Permutation tests (n = 200) for validation of the PLS-DA models comparing (a) CP24 vs C24 and (b) CP24 vs P24.** Purple and orange bars represent the frequency distributions of Q² and R²Y values obtained from permuted models, respectively. Arrows indicate the R²X, R²Y, and Q² values of the original models. Empirical permutation p-values for R²Y and Q² are shown in each panel.

**Supplementary Table**

**Table 1 The contents of volatile compounds of different fermented HBB (n=3)**

| **Compound** | **content (μg/kg)** | | | |
| --- | --- | --- | --- | --- |
|  | **CK** | **C24** | **P24** | **CP24** |
| **Ketones** | **19631.29±77.80** | **23843.62±923.56** | **23553.38±1883.44** | **25847.97±672.55** |
| 2-Cyclohexen-1-one, 3-methyl- | 3701.52±106.10 | 3641.69±46.25 | 4111.59±351.61 | 3815.47±80.84 |
| 2-Cyclohexen-1-one, 4-(1-methylethyl)- | 2873.06±34.92 | 3072.83±50.17 | 3471.62±300.02 | 3433.12±63.80 |
| 2,6,6-Trimethyl-2-cyclohexene-1,4-dione | 2617.24±28.22 | 2679.02±61.20 | 3028.01±287.58 | 2904.25±55.16 |
| 4-Methyl-2-oxetanone | 65.42±44.62 | 3252.80±502.56 | 1320.52±101.52 | 3429.27±473.35 |
| Furaneol | 1072.09±28.17 | 1076.89±20.28 | 1210.73±118.94 | 1117.27±12.86 |
| 1,3-Cyclohexanedione, 5,5-dimethyl- | 868.32±16.30 | 1000.23±33.21 | 1047.26±94.45 | 1097.09±32.37 |
| 3(2H)-Furanone, dihydro-2-methyl- | 1332.38±34.93 | 628.10±64.52 | 925.10±93.75 | 573.67±81.78 |
| 2(5H)-Furanone, 5-ethyl-3-hydroxy-4-methyl- | 695.79±8.55 | 771.37±19.84 | 833.03±70.81 | 850.62±21.84 |
| Cyclohexanone, 5-methyl-2-(1-methylethyl)-, (2R-cis)- | 705.09±6.88 | 702.63±13.68 | 790.76±75.57 | 749.31±11.89 |
| 5-Nonanone | 590.00±22.45 | 611.66±9.12 | 689.13±61.83 | 658.56±16.54 |
| 1,2-Propanedione, 1-(2-thienyl)- | 544.81±10.22 | 553.38±14.23 | 643.85±54.58 | 605.83±17.01 |
| 3-Decen-2-one | 541.90±9.52 | 549.37±14.44 | 638.25±54.05 | 601.17±17.60 |
| 4-Heptanone | 450.41±9.27 | 468.18±5.56 | 475.94±19.53 | 517.02±5.77 |
| Ethanone, 1-(2-hydroxyphenyl)- | 398.83±0.91 | 407.08±10.56 | 453.83±43.47 | 439.56±7.41 |
| **Terpenes** | **18893.46±118.68** | **20135.30±600.41** | **21841.24±1838.69** | **22181.83±309.07** |
| Neral | 3998.01±72.64 | 4029.80±124.38 | 4658.56±400.89 | 4358.62±132.70 |
| 1,7,7-Trimethylbicyclo[2.2.1]heptan-2-ol | 1749.03±12.46 | 1753.42±43.72 | 1967.27±186.26 | 1869.53±28.42 |
| endo-Borneol | 1747.61±12.50 | 1750.88±44.03 | 1965.71±186.37 | 1867.76±28.16 |
| Bicyclo[2.2.1]heptan-2-ol, 1,7,7-trimethyl-, (1S-endo)- | 1747.61±12.50 | 1750.88±44.03 | 1965.71±186.37 | 1867.76±28.16 |
| 6-Octen-1-ol, 7-methyl-3-methylene- | 949.78±16.60 | 1086.21±35.26 | 1146.47±101.46 | 1179.00±46.75 |
| L-Menthone | 705.09±6.88 | 702.63±13.68 | 790.76±75.57 | 749.31±11.89 |
| Cyclohexanone, 5-methyl-2-(1-methylethyl)-, cis- | 705.09±6.88 | 702.63±13.68 | 790.76±75.57 | 749.31±11.89 |
| Cyclohexanone, 5-methyl-2-(1-methylethyl)-, trans- | 705.09±6.88 | 702.63±13.68 | 790.76±75.57 | 749.31±11.89 |
| 2-Methyl-6-methyleneocta-2,7-dien-4-one | 597.16±1.91 | 607.59±14.44 | 677.66±65.59 | 655.59±9.82 |
| Cyclohexanol, 2-methyl-5-(1-methylethenyl)-, (1.alpha.,2.beta.,5.alpha.)- | 314.97±6.26 | 485.62±35.09 | 385.72±35.16 | 521.51±34.98 |
| Phenol, 2-methyl-5-(1-methylethyl)- | 170.92±18.11 | 569.82±68.23 | 281.80±28.05 | 596.51±17.16 |
| Isopulegol | 340.67±2.99 | 338.51±6.45 | 382.48±35.82 | 364.00±5.25 |
| Bicyclo[2.2.1]heptan-2-ol, 2,3,3-trimethyl- | 338.12±3.02 | 336.92±6.62 | 380.42±35.65 | 361.88±5.11 |
| Thymol | 144.20±15.77 | 496.22±60.65 | 239.47±24.61 | 517.88±15.62 |
| Cyclohexanone, 2-methyl-5-(1-methylethenyl)-, trans- | 262.28±3.43 | 278.80±5.01 | 315.27±26.24 | 310.13±4.65 |
| 6-Octen-1-ol, 3,7-dimethyl-, (R)- | 226.54±4.56 | 230.28±5.82 | 266.94±22.36 | 254.67±5.62 |
| Citronellol | 226.54±4.56 | 230.28±5.82 | 266.94±22.36 | 254.67±5.62 |
| **Esters** | **13060.55±173.45** | **18481.11±1037.49** | **16836.17±1218.52** | **20181.74±998.92** |
| ethyl 3-octenoate | 2908.69±36.14 | 3108.52±51.82 | 3519.61±298.00 | 3473.00±64.04 |
| Bicyclo[2.2.1]heptan-2-ol, 1,7,7-trimethyl-, formate, endo- | 1266.18±19.58 | 1279.70±42.69 | 1481.71±128.59 | 1385.99±41.70 |
| Isobornyl formate | 1266.18±19.58 | 1279.70±42.69 | 1481.71±128.59 | 1385.99±41.70 |
| Propanoic acid, 1,1-dimethylethyl ester | 120.79±0.93 | 1499.28±205.25 | 881.17±328.70 | 1413.73±196.35 |
| n-Pentyl isothiocyanate | 917.46±3.44 | 901.21±25.80 | 1064.71±92.63 | 992.98±24.79 |
| Tetrahydrofurfuryl propionate | 615.69±2.78 | 624.56±12.98 | 698.29±67.88 | 673.88±11.77 |
| Ethyl octanoate | 2.97±0.98 | 1194.10±208.99 | 110.88±21.63 | 1231.63±261.36 |
| 4-Heptanol, 2,6-dimethyl-, acetate | 568.92±12.08 | 570.47±17.17 | 672.56±60.27 | 630.03±7.91 |
| Butyl angelate | 522.61±13.35 | 522.19±14.33 | 619.53±57.72 | 575.22±10.64 |
| cis-3-Hexenyl isovalerate | 408.95±7.04 | 413.42±11.04 | 480.91±40.80 | 449.91±12.66 |
| n-Valeric acid cis-3-hexenyl ester | 408.95±7.04 | 413.42±11.04 | 480.91±40.80 | 449.91±12.66 |
| cis-3-Hexenyl-.alpha.-methylbutyrate | 407.66±7.25 | 411.81±10.55 | 479.00±41.16 | 448.65±12.71 |
| 2-Octenoic acid, ethyl ester | 322.06±7.31 | 329.77±7.84 | 379.33±30.36 | 363.52±6.27 |
| Ethyl decanoate | 1.94±1.48 | 452.16±58.71 | 317.93±110.11 | 453.93±136.68 |
| 3-Octen-1-ol, acetate, (Z)- | 214.32±4.00 | 240.26±7.30 | 256.99±22.24 | 265.56±5.65 |
| Methyl methacrylate | 25.88±0.20 | 388.23±61.28 | 148.02±11.77 | 412.19±54.93 |
| Butanedioic acid, diethyl ester | 3.25±2.12 | 424.59±72.24 | 44.45±9.23 | 443.10±91.26 |
| Pentanoic acid, butyl ester | 178.18±4.76 | 180.67±8.90 | 211.13±19.54 | 197.40±2.04 |
| Methyl tiglate | 171.92±2.93 | 177.99±1.42 | 180.61±7.58 | 196.36±2.45 |
| 2-Pentanol, acetate | 161.55±4.86 | 168.82±0.97 | 169.60±6.37 | 187.74±2.33 |
| trans-2-Hexenyl isovalerate | 150.13±4.62 | 160.68±5.99 | 180.58±18.12 | 177.03±8.12 |
| **Aldehydes** | **11096.20±95.20** | **11978.93±263.40** | **12426.78±262.78** | **13003.09±88.13** |
| (E)-2-Hexenal | 2649.23±70.74 | 2746.42±17.58 | 2799.67±129.87 | 3059.73±25.61 |
| 2-Hexenal | 2647.34±71.01 | 2744.33±17.47 | 2797.83±130.14 | 3057.42±25.57 |
| 2-Heptenal, 2-propyl- | 1846.99±24.51 | 1957.59±48.99 | 2182.51±185.28 | 2127.09±48.50 |
| Cyclohexanecarboxaldehyde | 773.17±3.80 | 748.86±14.89 | 827.54±70.24 | 759.62±4.03 |
| Butanal, 3-hydroxy- | 503.36±4.29 | 500.51±5.60 | 497.38±1.01 | 500.66±6.73 |
| 6-Octenal, 7-methyl-3-methylene- | 310.02±1.26 | 323.69±5.49 | 357.50±34.71 | 356.03±5.51 |
| Acetaldehyde, (3,3-dimethylcyclohexylidene)-, (Z)- | 313.63±4.10 | 315.53±8.77 | 366.55±31.67 | 342.11±10.53 |
| **Alcohols** | **10508.24±68.73** | **30814.99±3548.27** | **18218.26±953.40** | **32882.64±2879.80** |
| 2-Pentanol | 397.20±3.05 | 5799.36±909.20 | 2252.59±174.13 | 6191.61±805.25 |
| 8-Azabicyclo[3.2.1]octan-3-ol, 8-methyl-, endo- | 2896.78±36.50 | 3089.29±50.97 | 3508.06±297.79 | 3458.88±64.75 |
| (S)-2-methyl-1-butanol | 324.47±2.49 | 4930.19±758.31 | 1838.35±131.31 | 5148.99±680.46 |
| 1-Butanol, 3-methyl- | 324.47±2.49 | 4930.19±758.31 | 1838.35±131.31 | 5148.99±680.46 |
| 1-Butanol, 2-methyl- | 324.47±2.49 | 4930.19±758.31 | 1838.35±131.31 | 5148.99±680.46 |
| Triethylene glycol | 913.37±4.38 | 921.34±16.31 | 1013.70±92.68 | 987.25±12.41 |
| 3-Quinuclidinol | 503.23±6.03 | 518.64±6.82 | 587.12±55.45 | 567.99±13.31 |
| Propylene Glycol | 493.31±3.33 | 489.50±1.11 | 491.45±1.01 | 490.38±0.61 |
| 3-Pentanol, 2-methyl- | 405.29±0.28 | 404.31±0.47 | 404.82±0.76 | 404.79±0.55 |
| Mequinol | 329.10±4.06 | 331.54±8.29 | 385.60±32.83 | 359.63±10.49 |
| trans,cis-2,6-Nonadien-1-ol | 311.91±1.59 | 325.96±6.09 | 359.39±35.03 | 358.21±4.78 |
| 2,6-Nonadien-1-ol | 311.91±1.59 | 325.96±6.09 | 359.39±35.03 | 358.21±4.78 |
| **Acids** | **3240.61±81.17** | **6092.35±388.91** | **4526.78±92.59** | **6486.59±254.32** |
| 2-Propenoic acid, 2-methyl- | 2088.22±131.75 | 2063.60±70.67 | 2013.67±117.26 | 2110.52±100.98 |
| Acetic anhydride | 126.16±136.06 | 2673.67±384.03 | 1235.37±86.62 | 2819.84±346.45 |
| 4,5-Dihydro-3-furoic acid | 317.22±4.77 | 320.73±10.32 | 372.67±32.09 | 347.70±10.28 |
| Butanoic acid, 2-hydroxy-3-methyl- | 102.65±0.93 | 99.51±1.03 | 112.76±9.45 | 102.97±1.74 |
| 2-Furanpropionic acid | 93.97±1.41 | 97.70±1.42 | 108.80±9.64 | 106.77±1.42 |
| 2-Methylbutanoic anhydride | 55.54±0.94 | 72.03±3.76 | 69.30±6.96 | 80.41±3.42 |
| **Ethers** | **418.01±4.42** | **1128.19±127.53** | **689.37±39.59** | **1203.58±93.30** |
| Ethane, 1,1-diethoxy- | 46.50±0.36 | 698.16±108.48 | 264.60±20.25 | 728.38±95.65 |
| 1,6-Octadiene, 3-ethoxy-3,7-dimethyl- | 208.05±2.30 | 214.16±5.50 | 239.83±24.43 | 234.75±3.94 |
| Benzene, 1,3-dimethoxy- | 35.87±0.63 | 36.56±1.30 | 40.37±3.65 | 39.26±0.24 |

Note: Contents were calculated using the internal standard method. Internal standard concentration = 10 μg/mL, added volume = 20 μL, sample mass = 500 mg; the equivalent internal-standard content was 400 μg/kg. Values are shown as mean ± SD (n = 3).

1. ^*^ Corresponding author at: College of Food Science and Engineering, Northwest A&F University, 22 Xinong Road, Yangling, Shaanxi 712100, PR China.

   *Email address:* dushuangkui@nwafu.edu.cn (S.K. Du).
